# Career Paths of Public Health Medicine Specialists in South Africa

**DOI:** 10.3389/fpubh.2019.00261

**Published:** 2019-09-12

**Authors:** Virginia E. M. Zweigenthal, William M. Pick, Leslie London

**Affiliations:** School of Public Health and Family Medicine, University of Cape Town, Cape Town, South Africa

**Keywords:** community medicine, preventive medicine, education, human resources, career paths, health services, public health

## Abstract

Public health (PH) skills are core to building responsive and appropriate health systems, and PH personnel including medical specialists are embedded in many countries' health systems. In South Africa, the medical specialty in PH, Public Health Medicine (PHM), has existed for over 40 years. Four years of accredited training plus success in a single national exit exam allows specialist registration with the Health Professions Council of South Africa (HPCSA). However, there are few posts designated specifically for PHM specialists in SA's health system. In view of uncertain roles, this research was designed to determine specialists' career paths, their work, job satisfaction, and perspectives on the future of the specialty. We combined three databases to generate the study population and invited all specialists to participate in an online or hard-copy survey. We found that in 2010, PHM was a small specialty of less 200 physicians. Of the 151 contactable, eligible physicians, 55.6% completed the questionnaire. Participants represented an aging group (median age = 49) of specialists and recent graduates were increasingly women. They largely worked in academic institutions (as managers, teachers, and researchers) and in the public sector health system; were motivated by a sense of social justice and their training was formative, exposing them to work settings which they later entered; were largely highly satisfied at work, but many worked in non-specialist positions. Indeed, one fifth had not registered with the HPCSA as specialists. They were concerned about the specialty's poor visibility and identity, but did not see other PH professionals as a threat. They believed that the specialty should refine its competencies, demonstrate its value and advocate for service positions at all levels of the public sector health service. PHM has a contribution to make—reorienting services to protect communities, preventing ill health, analyzing disease burdens locally, identifying innovations in a resource-constrained health service, largely preoccupied with curative care services.

## Introduction

Public health (PH) skills are core to building responsive and appropriate health systems. PH approaches are required to support transformation of inequitable health systems such as SA's apartheid health system to improve health outcomes. This is reflected in key current health policy documents in South Africa (SA) related to Human Resources for Health (HRH) (1), the National Health Insurance (NHI) White Paper (2), and the National Public Health Institute of South Africa (NAPHISA) Bill (3).

PH personnel, including medical specialists, are embedded in many countries' health systems. In SA, the specialty of Public Health Medicine (PHM), then called Community Health, was established in 1975 to create a cadre of doctors who could hold senior management and leadership positions in the health sector in national, regional, and metropolitan areas (4). Modeled on the British specialty, the service role was expanded to include academia, research, health policy-making, and leadership that aimed to improve the health status of populations (4).

During SA's apartheid era until 1994, preference for the appointment of the officer responsible for environmental health, communicable disease control, and health promotion in local authorities was a medical practitioner with some form of community health qualification such as a Diploma in Public Health (DPH) (5). Some incumbents held PHM specializations which provided broader competencies than were required by Medical Officers of Health (MOHs). Health departments headed by such MOHs existed in most large cities. However, this position disappeared in in the post-apartheid era along with a shift away from medically trained hospital managers (6).

Fellowships of the College of Public Health Medicine (CPHM) or before 2010, Masters in Medicine (MMed), enabled registration as a Public Health Medicine or Community Health specialist with the Health Professions Council of South Africa (HPCSA). The CPHM is a constituent college of the Colleges of Medicine of SA, the body empowered by regulation to conduct examinations for medical specialists nationally. MMeds are academic qualifications for specialists conferred by universities accredited to train specialists. However, anecdotal information suggests that not all those qualifying as PHM specialists between 1999 and 2009 had registered as specialists.

While PHM and PH skills sets are acknowledged in current HRH policy in SA (1), this has not translated into a career path for PH professionals and PHM specialists in SA. Consequently, their specific intended roles and career paths are uncertain.

Only one employment option is unequivocal: employment in “joint” academic posts for training physicians in PH at both undergraduate and postgraduate levels. These joint posts must be filled by PHM specialists under a partnership between universities and provincial departments of health. “Joint appointment” staff are usually formally employed on provincial conditions of service with clinical responsibilities in provincial health services and teaching and research responsibilities linked to the university.

In 2009, SA introduced a strategy to attract and retain physicians and specialists in the public sector—the Occupational Specific Dispensation (OSD) (7), which introduced new salary scales for all specialists, including PHM specialists. However, the OSD excluded PHM specialists working in management posts, as these jobs fell into management salary scales.

Other than university academic posts and a limited number of PHM specialist posts in two provinces (8, 9), no health services posts currently require a specialist qualification in PHM. In this context, we set out to enumerate and describe the work of PH specialists, their motivations for specialization, job satisfaction, and their perspectives on the future of the specialty and training programs. While specialists-in-training (called registrars in SA) were motivated by a desire to improve SA's health system and communities' health status (10), it is not known what specialists overall motivations for practice are.

Our intention was to use this information to contribute to the design of the health workforce for the SA health system in a period of health sector reform toward the NHI, focus training curriculum discussions of the schools of PH, academic departments of PHM and the CPHM, and contribute globally to information and debates about roles of physician specialists in PH. Data for this article were collected by the first author for her doctoral work completed in 2015 (11).

## Methods

In 2012, we conducted a cross-sectional analytical study, surveying all physicians who had successfully exited PHM training programs in SA, from the inception of the specialty until December 2010. A challenge was to identify people who had completed the training, as not all had registered as specialists with the HPCSA, and university-held databases did not always note if registrars successfully completed training. Our sampling frame was a database created from three possible sources of data: (1) the HPCSA specialist register of Public Health Medicine or Community Health to the end of 2009; (2) members of the CPHM before August 2010; and (3) graduates of accredited university training programs with MMed degrees. The CPHM database was taken to include that of its predecessor bodies (the Faculty of Community Health and the College of Community Health). Additionally, two types of CPHM members exist—fellows who have passed the CPHM exams, and associates, who entered the College with completed university MMeds and were recognized as having specialist competencies by their peers in the CPHM, through an application-nomination process (12). Both associates and fellows were included for this study.

We excluded “founder members” of PHM who, as elsewhere (13), were “grand-fathered” into the specialty. Occupational Medicine (OM) specialists, who qualify through examinations administered by the OM division of the CPHM, were also excluded as their scope of practice is chiefly clinical. The dataset included physicians' birth dates, sex, years when qualified as physicians and specialists, and the universities where they qualified. Only 177 potential specialists were identified. Most (78.5%) were members of the CPHM, 59.9% were registered as specialists with the HPCSA and 30.5% had MMed degrees. Only 19 (10.7%) were all three: were on the CPHM database, held MMed degrees, and were registered as specialists. Of CPHM members, 42% (58/139) were not on the HPCSA specialist register.

**Figure 1:**
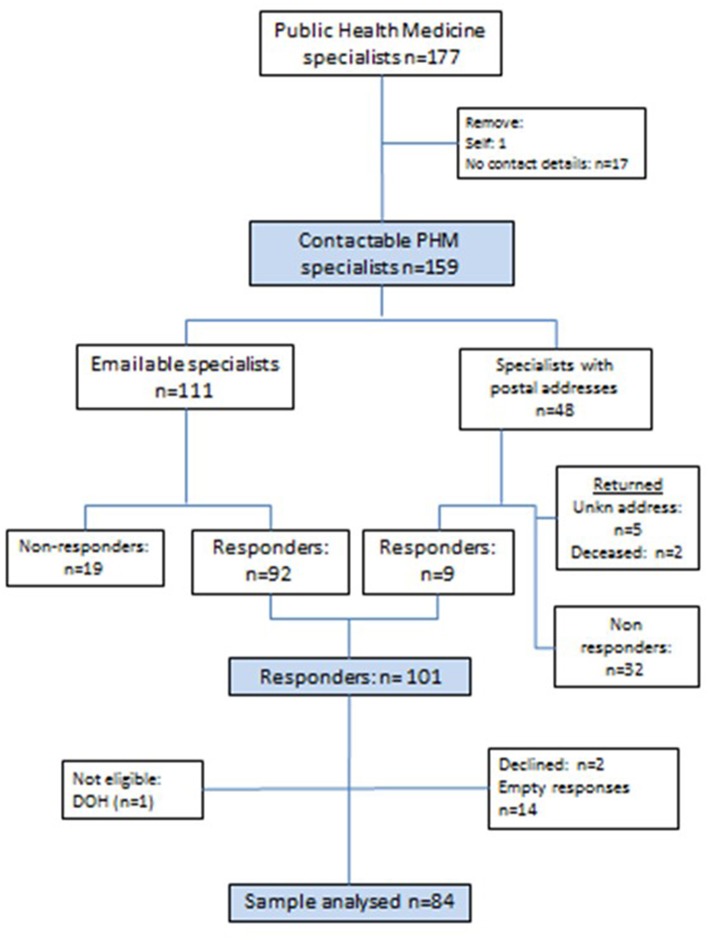
Flow diagram describing eligibility and respondents.

The derivation of the final sample for the study is described in Figure 1. After removing the first author and CPHM members with no contact details (*n* = 17), 159 physicians were invited to participate. Those with email addresses (*n* = 111) were invited to complete an online questionnaire (see Datasheet 1), and those with only postal addresses on the HPCSA register (*n* = 48), were sent stamped and addressed return envelopes inviting participation. Seven letters were returned unopened: two noted that the addressee was deceased and five were returned as an “address unknown.” A further 19 did not respond to email invitations leaving 101 who responded. Of these, two declined participation and one non-eligible respondent (not on the HPCSA register as a specialist) were removed from the database. Therefore, two thirds of those approached (101/151) responded and 84 (55.6%) completed the questionnaire.

### Research Tool, Enrolment, and Analysis

The study tool was a self-administered online questionnaire with closed- and open-ended items, using SurveyMonkey™ that allowed online development, administration, and data-entry of questionnaires. All participants were first emailed, informing them about the study and inviting future participation. Two weeks later, emails launching the study with the link to the SurveyMonkey™ site were sent and participants were given 3 weeks to complete the survey. Non-responders were identified from a software function that tracked response rates and submissions, and two reminders were sent, which extended the deadline by 6 weeks. The survey is included as additional material. An Excel spreadsheet for the electronic survey was downloaded from SurveyMonkey™. Data for the nine who responded to the postal survey were double entered manually onto the spreadsheet.

Responses were secured in a password protected file, and we removed any personal identifiers. The cleaned, quantitative data were analyzed using STATA®13. Descriptive analyses and differences in responses by demographic and other variables are reported, at *p* < 0.05 for levels of significance. *T*-tests and Mann-Whitney tests were performed for normally distributed and skewed numerical data respectively and, chi-square or Fisher-exact tests, for categorical data. Linear regression for continuous, and Poisson regression for count data were undertaken. Post-coding was conducted, re-categorizing data for demographic factors, career choices, registrar training, and jobs (employers and roles). Qualitative information from the open-ended questions was entered into Atlas ti7; content analysis was performed on these responses; and quotations, illustrating themes, were selected. Demographic descriptors for respondents in quotations are noted by sex and age (sex, age). F is a female, M is a male, and age is given in years.

### Validity

As no identifiers for respondents were captured, they could not be compared to non-respondents. Instead, to ascertain if respondents differed from specialists on the database, their demographic and training data were compared to those on the composite database. As continuous data were normally distributed, single sample *t*-tests and chi-square tests for proportions were performed, using *p* = 0.05 as the level for significance.

### Ethics

This study was approved by the University of Cape Town's (UCT) Faculty of Health Sciences Human Research Ethics Committee (Ref 410/2010). Participation in the survey was entirely voluntary and consent was obtained. A consent narrative served as an information sheet in SurveyMonkey™, outlining the purpose of the survey and participants ticked a box that noted questionnaire completion indicated consent. We assured participants that confidentiality and anonymity would be maintained, that participation did not require response to all questions and that they could withdraw from the study. For postal responses, all respondents signed and returned the consent form which acknowledged autonomy, confidentiality, and anonymity. No incentives were offered for participation. Names were not linked to data and so respondents remained anonymous. The database containing the raw online submitted data was accessible only to the first author.

## Results

The survey had a completion rate of 56% (84/151). Respondents did not differ from those in the composite database in terms of sex [female: 46 vs. 41% (*p* = 0.34)] or under- and post-graduate training institutions, but on average they qualified as physicians 3 years later than the overall population [1986 vs. 1983 (*p* = 0.01)].

### Demographic Characteristics and Educational Backgrounds

**Figure 2:**
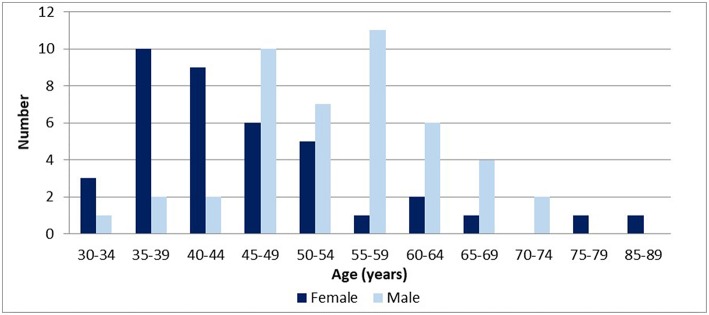
Age of respondents by gender (*n* = 84).

The median age of respondents was 49 years (IQR: 42–58 years); and 46% were female. Males tended to be significantly older than women [median age = 59 years (IQR:49–64) vs. 44 years (IQR:39–54); *p* < 0.001]. Women completed specialist training more recently than men, and differences were significant [median year for women being 2004 (IQR: 1997–2009), vs. 1993 for men (IQR: 1989–1999); *p* = 0.00]. The distribution of respondents' age is presented in Figure 2.

Over three quarters of respondents (81.0%) were born in SA, 10% were born elsewhere in Africa, and 8% in Europe. Most (74/84) did their medical training in SA, and 7% trained in Europe. The median year for completing physician training was 1986 (IQR: 1980–199; *n* = 74). Of the 88% completing their medical training in SA, most (39%) trained at UCT, followed by 22% at the University of the Witwatersrand (Wits), 14% at University of Pretoria (UP), and 12% University of KwaZulu-Natal (UKZN). A number of respondents (30% or 25/83) had obtained other tertiary qualifications prior to specializing, mostly in PH related areas, such as diplomas in occupational health (DOH) (8%) or in tropical medicine and hygiene (DTM&H) (8%).

### Training Experience and Competencies

Specialist training experiences were explored as these was likely to have an effect on subsequent career trajectories, and influence opinions about the value and future of the profession.

The median year that respondents started specialist studies was 1994 (IQR: 1987–2003; *n* = 83), and the median time from completing their medical qualification to specialist training was 7 years (IQR: 5–10). Respondents completed specialist training at UCT (38%), followed by Wits (29%), UKZN (13%), UP (7%), Stellenbosch (7%), and Medunsa (4%). The distribution of their medical training by institution was similar.

Their 4-year training comprised theoretical and experiential learning in range of service settings. The median number of rotations was four (IQR:3–5) and placements depended on their funding. Registrars are usually employed by provincial departments of health, and 66% of respondents had such posts. However, some (6%) were provincially employed medical managers of hospitals or program managers and others were researchers (12%) working at universities or the Medical Research Council (MRC). Those not in salaried provincial registrar positions continued to work for employers.

Work placements, called rotations, changed over time. Rotations were in service settings, and mostly in provincial health departments, health districts, and hospitals. Early trainees' service experiences and rotations were unstructured whereas more recent ones were more formalized but depended on the availability of hosts. The geographical proximity of research institutions such as the National Institute of Communicable Diseases (NICD) and the SA Medical Research Council MRC affected rotation options.

**Figure 3:**
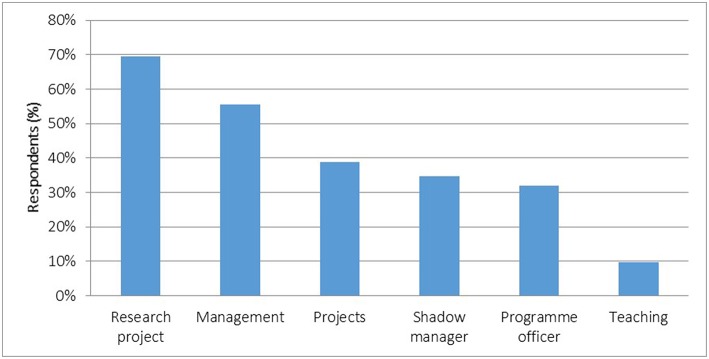
Work performed in training rotations (*n* = 72).

Respondents reported working in a variety of roles as registrars (Figure 3). Most (56% or 40/72) were managers within organizations, in health programs and managed projects allocated to them. A third shadowed managers with little responsibility. Some (10%) taught undergraduate medical students. Most (69%) conducted service-related research projects.

Competencies developed through placements were in communicable disease control (57% or 40/73), policy and planning (46%), and monitoring and evaluation (43%). They mostly acquired research and management skills in hospital management (43%), information management (22%), and program or project management (27%).

Many (47% or 37/79) wanted additional placements to acquire skills for future careers. These clustered in competencies obtained by other registrars in rotations they missed, and in occupational and rural health. They wanted work experience at all levels of the health system: in local authorities, district, provincial, and national as well as international placements such as the World Health Organization (WHO).

Course work is prominent in registrar training, and they all participated in postgraduate courses through attending lectures, completing course assignments and, in some cases, obtaining postgraduate degrees. Courses followed varied by institution and some institutions awarded registrars' additional degrees for courses passed. Overall less than half were awarded additional qualifications as this was viewed to be part of registrar training. Most (46%) completed the requirements for diplomas in occupational health and health management (31%), followed by 30% who completed Masters of Public Health (MPH), and 17% completed diplomas in tropical medicine and hygiene (DTM&H).

Apprenticeship-type training, the specialist training model, should involve mentorship guiding training. Over 70% (57/80) had mentors and of these, 72% (41/57) rated them as good or outstanding but 9% (5/57) rated them poorly.

Many (44% or 37/84) identified specific placements that gave direction to subsequent careers. The most common formative experiences were in communicable disease control (27%), health planning (19%), monitoring and evaluation (M&E) (16%), hospital management (14%), and research projects that informed later research careers (14%). More women (19/39 or 49%) than men (18/45 or 40%) felt that specific training exposures gave direction to their careers but this difference was not statistically significant (*p* = 0.422).

### Registration of Qualification and Specialty

Post examination, HPCSA specialist registration takes place in two steps. The additional qualification must first be registered, followed by application for specialist registration. Respondents were asked if they had registered their additional qualification and their specialty, and reasons were probed.

**Figure 4:**
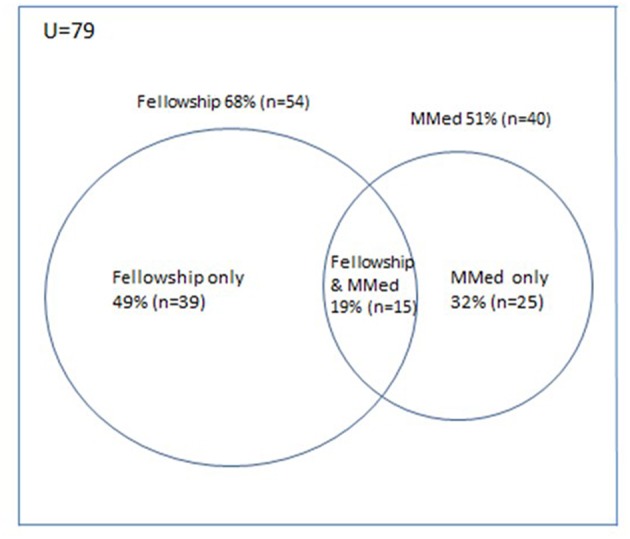
Specialist qualifications obtained.

Most respondents (98% or 82/84) specified their specialist qualifications: 79 obtained either an MMed or CPHM fellowship. For two, a diploma and a doctorate allowed specialist registration, but one on the CPHM database could not register as the HPCSA did not recognize the UK PH qualification. Most (68%) obtained a CPHM fellowship qualification and 51%, an MMed (Figure 4). A minority (19%) obtained both the fellowship and MMed. Most specialists (79%) who trained through traditionally English-speaking universities, obtained a CPHM qualification, which was significantly higher than the 27% obtaining the CPHM qualification from traditionally Afrikaans-speaking universities (*p* = 0.0017).

Ninety percent (74/82) registered their additional qualification, and of these, 85% (63/74) registered as specialists. Of those who obtained either an MMed or CPHM fellowship qualification, 20% (16/79) did not register as specialists. The proportions of those registering as specialists did not differ by age or time since qualifying. Reasons for specialist registration included professional recognition or meeting a job requirement for a service or academic post. Some waited to register until the salary advantage of the 2009 OSD in the public sector became apparent. Reasons given for not registering, were that it was not a requirement for a position (*n* = 3), ignorance about the process (*n* = 2), cost incurred in the face of no benefit (*n* = 1), belief that only an MMed was required (*n* = 1), difficulties with the HPCSA process (*n* = 1), and emigration (*n* = 1).

### Career Trajectories

After specializing, respondents had long careers—a median of 15.5 years (IQR:6–20), and on average they had three jobs (IQR: 2–4; range:1–6; *n* = 80). Men reported significantly longer career trajectories [median = 19 years (IQR:13–23)] than women [median = 8 years (IQR:3–15)] (*p* < 0.001), which is consistent with women being younger and more recent qualifiers. Further analysis showed that career length was related to respondents' age (*p* = 0.00) and while sex had an effect, this it was not statistically significant (*p* = 0.14).

**Figure 5:**
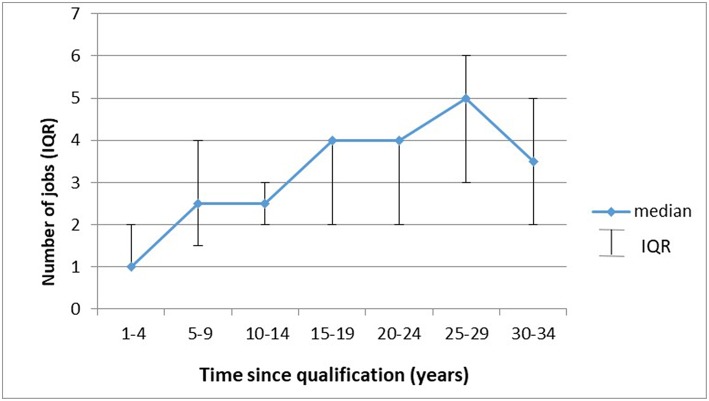
Number of jobs over time since specialization (*n* = 80).

Figure 5 illustrates that the longer respondents had been qualified the more jobs they were likely to have had. Specifically, their median number of jobs increased with time since qualification (*p* < 0.001), and after 10 years, 30% were likely to change jobs (95% CI: 14–42%). Nonetheless, a sizeable proportion (21%) only ever had one job. Some respondents (32% or 26/80) changed positions with the same employer. Specialists in provincial departments of health were employed in various capacities over time. While men had significantly more jobs than women [mean = 3.4 (sd = 1.55) vs. 2.4 (sd = 1.48); *p* = 0.004], linear regression showed that time since qualification (*p* = 0.02) rather than gender (*p* = 0.20) impacted on number of jobs.

### Work History in Academic and State Health Services

Most respondents had been employed, at one time, by universities (55% or 44/80) or in the state health sector (63% or 50/80) at all service levels: 56% had worked for provincial departments of health, 18% for local authorities, and 8% for national government. Of the ten specialists who emigrated, four became academics, three worked for international NGOs, two worked as health managers, and one was a clinician.

**Figure 6:**
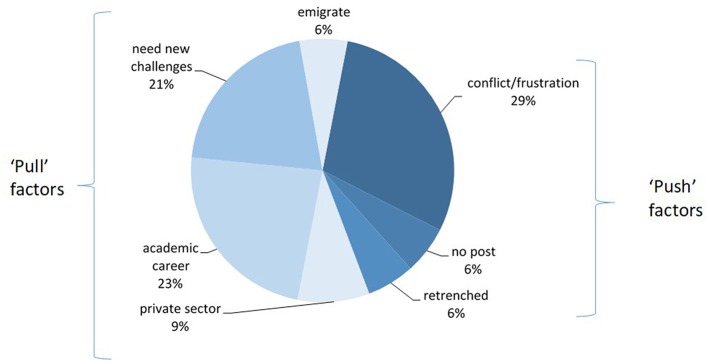
Reasons for state-employed specialists leaving the state health sector (*n* = 36).

Most respondents (70% or 36/50) who had ever worked in SA's state health sector had since left that employment. Reasons for leaving state health sector work, in Figure 6, included “pull factors” such as new “challenges” or career opportunities, such as academia, relocation elsewhere in SA or abroad, and moving into the private sector. “Push” factors were unhappiness at work due to conflict or frustration, and being forced to leave (retrenched, restructured out of a job, or the unavailability of a specialist position).

### Work in 2012

**Figure 7:**
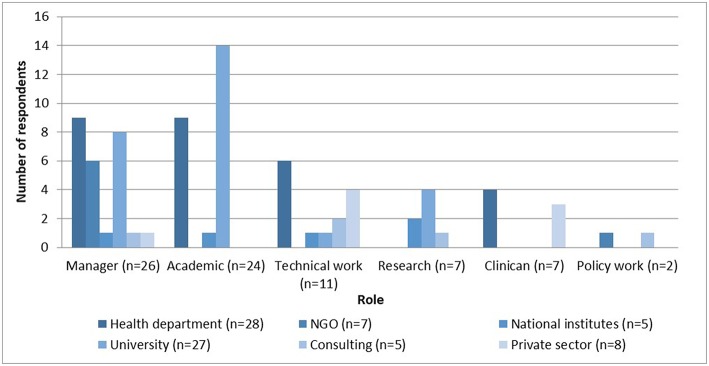
Roles of specialists, by employers (*n* = 80).

Respondents' employers and roles in 2012 are described in Figure 7. Most (35% or 28/80) were employed by government health departments, with a third (9/28) working in “joint appointment” positions; followed by universities (34% or 27/80), both local and international; NGOs (9%) and the SA MRC; or the National Institute of Occupational Health (6%). For this analysis, “joint staff” were classified as being employed by provinces, although they have academic as well as service responsibilities

The most common roles performed in organizations were managerial (33%), then academic (teaching and research) (30%), technical work (18%), pure research (7%), and direct patient care (7%). Managers mostly worked in departments of health (35%), in university management (31%), and NGOs (23%). Most academics worked for universities (58%) and a further 38% worked for departments of health in “joint appointment” positions. Technical work—designing or implementing policies and programs—was specialists' work in health departments (43%), the private sector (29%), or in consultancies (14%). Pure researchers worked in universities (57%), the MRC (29%), or as consultants (14%). Most clinicians worked in the state sector (57%). Policy work was done by specialists working in NGOs or through consultancies.

No statistically significant differences were detected in whether HPCSA registered specialists had different career destination compared to those who did not register.

### Further Education

Many respondents (45% or 37/83) pursued further degrees after specialist qualification: 27% (10/37) completed PhDs and a further 19% were completing one at the time of the survey. Other further qualifications include Masters in Business Administration (MBA) (4/37), economics qualifications (3/37), and psychiatry (3/37). Most academics or researchers (66%, 19/29) were 3.42 (OR CI: 1.14–10.45) times more likely to have completed higher studies than other specialists.

### Job Satisfaction

Most specialists rated their careers highly, with 41% reporting that they had “found their niche,” and 23% being “very pleased.” Those who “found their niche” enjoyed their work and felt that they did it well. More men (68%) were “very pleased” or had “found their niche” than women (58%), but this difference was not statistically significant (*p* = 0.29). Respondents over the median age of 49 were significantly more likely to rate their careers highly (76%) compared to respondents 49 or younger (53%) (*p* = 0.035).

Job satisfaction was analyzed by employer and role at work (Table 1). Most (57%) who worked for departments of health were “very pleased” or had “found their niche.” No particular employer was associated with a specific rating in job satisfaction, except for those working in NGOs, who were all satisfied or more. Most managers (72%) and academics (55%) were “very pleased” or had “found their niche.”

**Table 1:** Respondents' job satisfaction, by employers and work roles (*n* = 78).

		**Not met expectations** **(*n* = 4)**	**Ambivalent** **(*n* = 10)**	**Satisfied** **(*n* = 14)**	**Very pleased** **(*n* = 18)**	**Found my niche** **(*n* = 32)**	**Total**
Employer	Health department (*n* = 28)	7%	18%	18%	11%	46%	100%
	NGO (*n* = 7)	0%	0%	14%	57%	29%	100%
	National Institute (*n* = 5)	0%	20%	20%	20%	40%	100%
	University (*n* = 26)	4%	15%	27%	19%	35%	100%
	Consultancy (*n* = 5)	0%	0%	0%	20%	80%	100%
	Private (*n* = 7)	14%	0%	0%	57%	29%	100%
Role in organization	Academic (*n* = 24)	0%	25%	21%	13%	42%	100%
	Manager (*n* = 25)	8%	8%	12%	40%	32%	100%
	Policy (*n* = 2)	0%	0%	0%	0%	100%	100%
	Research (*n* = 7)	0%	0%	57%	0%	43%	100%
	Clinician (*n* = 6)	17%	17%	17%	17%	33%	100%
	Technical work (*n* = 14)	7%	7%	7%	29%	50%	100%
	Total (*n* = 78)	5%	13%	18%	23%	41%	100%

All those who had emigrated rated their careers highly, and moved to advance academic and international careers, or because of perceived shrinking SA job opportunities.

The 18% (14/78) who were “ambivalent” or whose careers had “not met expectations” gave reasons related to work environment (such as “lack of collegiality”), or not having found work they had hoped for (such as being a “technical expert” in the services and research), or being too early in their career. A few women cited life circumstances, such as working part-time to accommodate child-care, which limited their job options.

### Motivations for Careers

Nearly all (95%) identified registrar experiences as being important in shaping their careers. Training gave skills, for example, management, planning, policy making and leadership skills, and research expertise which were valuable for later work. Exposures motivated some to “contribute to the public sector health services” and others, to work in academic environments doing “clinical and epidemiological research.” Often, these exposures opened academic research opportunities.

In addition, values, lifestyle, and professional relationships attracted respondents to work opportunities, with the dominant themes being commitment to equity and social change, valuing family life, mentors and colleagues, as well as content interests. Their commitment to social justice and improving the health status of populations translated into careers to ensure equitable, quality health services. Some took up opportunities in interest areas, such as communicable disease control, the HIV epidemic, and management. For many, job security and commitment to a quality family life were important, while others highlighted inspiring mentors, a good working environment, and colleagues.

My experiences as a student—propelled me into the social justice field. My work following graduation as a doctor, similarly, directed me to social justice, but also to a public health career (M, 51).

[My] desire [was] to contribute to positive change in health care delivery and health outcomes through policy and health systems reform (F, 37).

I try to work for people who I can respect and on issues which I think are important (M, 48).

### Prospective Work in the State Health Sector

In light of current health service reform in SA and the need for PHM specialists made in policy documents, respondents' perspectives on work in the public sector were probed. Many local respondents (35% or 25/72) already worked in departments of health. Of the 40 “working age” respondents (<65 years) employed outside the public sector, 20% (8/40) would “definitely” consider moving to the public sector, 60% (24/40) might consider this, and 18% (7/40) would never consider such a move.

While over a fifth (23%) believed that the salary advantage of the OSD would attract specialists to the public sector, most (56% or 45/80) were unsure of its effect, and 20% did not believe it would attract specialists. Respondents believed the OSD made salaries competitive, gave parity amongst specialists, and would attract people to specialize, rather than complete MPHs. On the other hand, those who did not believe this salary incentive would attract specialists to the public service, felt that work environments, critical for recruitment, and retention, were not optimal in the state sector and argued that specialist qualifications were seldom required for management posts. Some believed that the few posts in the services—a diminished demand—were due to a lack of clarity regarding specialist roles.

I do however think that the OSD will not necessarily attract PHM specialists into the public service because of the lack of clarity regarding the role of PHM specialists in the health system; and other factors beyond the lack of financial incentives (e.g., inefficiency, bureaucracy, poor leadership) also prevent doctors from joining the public service (F, 44).

For some, PHM specialists were disadvantaged compared to other medical specialists as there are mostly no overtime options, salary scales are lower and the potential for advancement is minimal.

I don't think that the incentives for PHM specialists match those of clinical specialists as there is less opportunity to have the equivalent positions e.g., “Head of a Clinical Unit”—and due to the reduced overtime payment for PHM specialists (F, 41).

### PHM Specialists and Other Public Health Professionals

**Figure 8:**
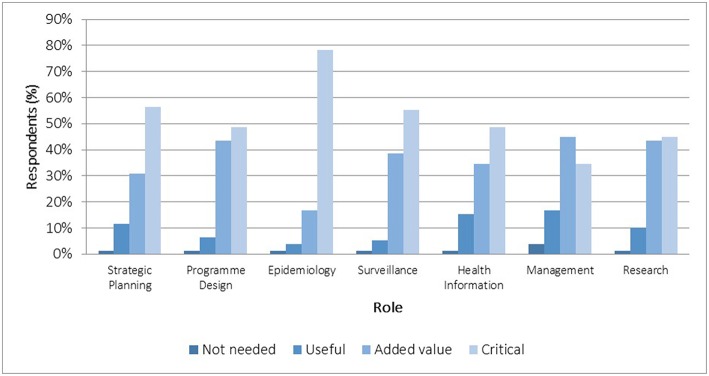
Respondents' perceptions of the importance of PHM specialists—by role (*n* = 78).

We probed perceptions about the value of specialists in seven domains of PH practice: epidemiology, surveillance, strategic planning, program design, management, health information, and research. In SA's health system, roles in planning, management, and health information analyses (using epidemiology and surveillance skills) are commonly performed by non-specialists. There was universal agreement that specialists were needed for all domains. Most perceived them as critical or adding value in measurement sciences (surveillance, epidemiology) and strategic planning (Figure 8).

Health service management was a contested area. Most believed they “added value” as managers, and some commented that senior health managers should be PH trained physicians or specialists. As this is not a requirement, the value and future of the specialty was questioned by some:

Unfortunately PHM specialists were replaced by all sorts of people with little/no knowledge of management [and] planning, leading to loss of interest of doctors to specialize. [The] MBA qualification is in demand in [the] private sector for administrative posts and public sector appoints politically correct candidates regardless of skills/qualifications (F, 46).

### Comparing PHM Specialists With Other Professionals

Many remarked about an overlap in competencies between PHM specialists and other PH trained professionals, believing that these professionals have important roles. Some added that personnel with MPHs and/or MBAs could perform many roles as well, or better in areas such as operational management, technical epidemiology work, and managing health information systems.

They add value by their individual inputs e.g., MBA will not give value to surveillance. MPH is usually program specific e.g., MPH health measurement or health systems excludes communicable diseases cadre (F, 52).

I think a well-qualified doctor with a thorough grounding via an MPH could do most of these with the relevant experience behind them (M, 56).

A few believed there were no roles that specifically required a PHM specialist. However, many volunteered that the difference between PHM and other PH trained professionals was the unusually wide range of knowledge and competencies that specialists have overall which yields a high value individual. High functioning professionals with broad skills are able work at senior levels in the health system. This was due to the mix of theoretical training and practical experience enabling mastery of skills, benchmarked against passing a nationally accredited exit examination:

In my view and experience, public health specialists with 4 years training and extensive service learning—which MPH students do not have—generally clearly outperform all others in the field in a range of critical performance areas e.g., critical analysis, cognitive depth, problem solving, innovation, planning (M, 57).

What the specialist qualification does is deliver someone immediately post-training with all the necessary qualifications and experience due to the rotations. So, it is a better package, less dependent on the individual career path following [the] MPH (M, 56).

The national college exams ensure that all specialists have the same exit exams, which does not happen with other qualifications (F, 35).

Given that only graduates with medical training can specialize in PHM, the value of a medical background was explored. Although individual patient care is not a PHM specialist competency in SA, many respondents believed that what is unique to the specialty is, through its population perspective, the ability to bring together clinical and population health skills. A medical background—understanding the biology of disease, and clinical experience—gave a deep understanding of health service issues needed for informed decision-making and insight for the design of programs to control diseases at a population level. In addition, some asserted that being a doctor facilitated communication with other physicians which commanded respect around issues of PH:

By virtue of their clinical training PLUS their PHM training, PHM specialists have an advantage over other public health professionals (even over doctors with an MPH) in terms of the skills-set that they bring to the health system, especially in areas such as strategic planning, health systems analysis and design of interventions, health systems, M&E (F, 44).

The clinical training gives them a specific advantage as they are able to communicate with other doctors in a language that they understand. The level of respect I command in a discussion with other doctors is different to that of a person with no undergraduate medical training. The undergraduate medical training gives us experience and specific insight into health care problems that may not be grasped by other cadres of workers (F, 35).

### Potential Roles for PHM Specialists in Current Health Sector Reform

Potential roles identified for PHM specialists in a reformed health service clustered around the versatility of PHM specialists and the need for competent managers. These included roles in health protection and promotion, in the management of complex programs, and supporting intersectoral interventions, which respondents argued PHM specialists are well-equipped to manage. Management roles identified included leadership at all levels of the health system, where PHM specialists would drive strategic planning, priority setting, and implementation.

Infectious disease programs require both clinical and epidemiological and management skills; in managing complex programs where services, personnel, and health programs are involved… Intersectoral work, for example, would be best done by a PHM graduate working in or leading a team (M, 51).

Other roles named included strengthening health systems and research. These were identified because respondents believed PHM specialists were trained in leadership, communication, and writing. Some raised challenges related to delegated authority and that specialists' roles should not merely be advisory, as had often been their experience.

Most commented on the lack of clarity about roles for PHM specialists in national policy. Many believed jobs should be in district health management teams as they could provide the technical skills for planning and management at this level and strengthen systems.

There is a need for proper strategic planning at district level, informed by collected data and planning. Efficient district health management will alleviate the resource needs at higher levels.… PHM should function as technical advisors at the district level. Having all categories of PHM—head of clinical Unit, specialist, and registrars including research assistants—will add value to research, policy, planning in districts (F, 52).

### Recommendations for the Future of the Profession

Most respondents (69%) commented on future PHM specialists career paths and identified the lack of clarity regarding its contribution in relation to other PH trained professionals as a threat to the specialty's future. Some recommended a process to reach clarity. First, the profession should be discussed with a range of role-players—within the profession, with registrars, program directors at universities, and employers such as departments of health, PH agencies, and regulatory bodies. This would result in a reformulated scope of practice, a revised curriculum, and training settings for future specialists. The profession should then be marketed and positions created in the public sector:

This conversation is long overdue and should also involve all the other role players, especially registrars. When I asked about career paths in my registrar interview, I was simply told that “all… graduates have managed to get jobs.” That is an inadequate answer (M, 48).

National consensus should be reached between provincial DOHs, CMSA, HPCSA, and the PHM training institutions regarding: (a) what are the areas in which PHM specialists have a unique advantage? (b) how should training be tailored?…and (c) for what kinds of jobs are we training PHM specialists (so that it is clear what it is that sets PHM specialists apart from other public health professionals)… All public sector institutions… should then start thinking about how to set up posts to accommodate PHM specialists (F, 41).

Many felt that the profession was not visible in the public sector health service or understood by potential employers. Consequently, the profession's profile needed to be raised. As is clear for other medical specialties, its value-add to the services and wide skills base should be made explicit.

Ensure that the national perception is clear of the added value and expertise. No one doubts that an orthopedic surgeon is better to deal with such problems than a GP and that where this can be met this is preferred. The same needs to be understood regarding what a public health specialist has to offer compared to a large number of generalists or people with narrow skills (M, 57).

A better understanding of the specialty should improve employers' recognition of its value and result in job opportunities. Many called for the establishment of positions at all levels of the service, and some argued that senior posts should be earmarked for PHM specialists. This advocacy process would be aided by PHM registrar work raising the specialty's visibility.

Fulfilling managerial roles in hospitals, strategic positions in NDOH, gaining a presence in the district, making a public health footprint in the services, not just academia (F, 35).

Us(e) the registrar rotations effectively to demonstrate our value (F, 36).

### Implications for Training

A clearer scope of practice and career paths would affect training requirements—both theoretical and experiential. Respondents outlined content training, registrar placements (service settings for experiential learning), and tasks that would equip specialists for work. Many (57%) gave suggestions for registrar training that came from reflections on their own training and subsequent work experience. Comments clustered around acquiring core competencies, the importance of registrars having responsibilities “so that they learn actively and not passively” and for rotations to be flexible to accommodate registrars' interests and career plans:

Focus training in the specific areas that we feel we have critical skill once these critical skills have been identified (F, 51).

Uneven formal training was a concern with some suggesting standardized academic programs that should focus on competencies in measurement sciences. Some suggested that a prior MPH should be required which would enable more flexible registrar placements that could be geographically distant from universities. Others argued that academic programs should not dominate registrars' experiences.

Specific improvements suggested were in leadership and management training, exposure to intersectoral work, skills in health economics, research, and writing skills—which should all be practiced in service settings. Respondents argued that some service settings, based on historical arrangements, needed to be reviewed and discontinued, unless they were supervised opportunities for core learning. They emphasized the importance of academic and service supervision which would enable trainees to be focused and get the required experience.

In the past, rotations have been determined as much by departmental politics as by common sense. Registrars get stuck in useless rotations, waste their time…(M, 48).

Identify experts in specific fields of public health at different institutions and let registrars rotate where experts operate. Do not limit training at the institution where registrars are registered (F, 46).

Mentors should help registrars choose projects to ensure that they develop all the required competencies. If this is left to the registrar or the services, the registrar might be disadvantaged and used as an extra resource and not as a trainee (F, 38).

A few suggested additional public sector training settings outside urban centers, in other parts of Africa as well as in the private sector. In these contexts, registrars could participate in systems strengthening efforts and this would sensitize trainees to service delivery problems in underserved areas:

Place registrars in areas of identified need, identified by the Department of National Health, e.g., health management, poorly functioning health facilities, poor infection control etc (M, 53).

## Discussion

This study characterized PHM specialists in SA, providing insight into their professional life, motivations and career trajectories, and their perspectives on the future of the profession. No comparable literature was found on training and work trajectories of PHM specialists from LMICs.

The actual size of the SA PH specialist pool was difficult to determine as many specialists (20% of respondents) were not registered and there were also flaws in the HPCSA register with “active” members including many deceased physicians. Nonetheless, it is evident that, in 2010, the specialty in SA was a small group of <200 working specialists for a population of 50 million people (14). This makes SA's specialist pool far smaller than the estimated 382 Community Medicine physicians for Canada's 23 million people (15) found in 2004 (16), the 1,673 (17) for France's 66.6 million people in 2015 (18), and the 1,100 (19) on the 2012 UK register for 63.7 million people (20).

Our survey completion rate was 56%, and respondents' demographics largely reflect those of all PHM specialists. They are generally a mature group of physicians (median age = 49), with men older than women. Interestingly the aging PH doctor pool was also the impetus in SA in the 1970s (4) for the creation of the PHM specialty. An aging PH workforce is an international concern, highlighted in literature from the UK (21) and the USA (22), and the WHO foresees severe shortages in the PH workforce (23). In SA, unless there is an accelerated production of new specialists there are adverse implications for the longevity and growth of the profession.

Overall, fewer specialists were women but this was the converse for recent graduates who were predominantly female. This may reflect the general feminization of the medical profession in South Africa over the past decade (24, 25) and of public health specifically, with more women entering specialist training (10) and women physicians increasingly completing MPHs (26). The feminization of medicine is an international phenomenon both for general medical training (27) and for public health training, for example in Italy (28). The editor of the SA Medical Journal in 2011, commented that SA women physicians “gravitate toward the less well-remunerated, so-called “soft” primary care specialties such as family medicine, pediatrics, and public health” (27). Although characterizing these specialties as “soft” is pejorative, a demographic shift to a predominantly female specialty seems to be true for PHM in SA, as has also been found in the UK (24) and for PH professionals overall in the USA (25).

International evidence shows that women choose specializations such as Family Practice and Pediatrics. Although they are less likely to work excessive hours, they are more likely to include more preventive care in their practice (29). These factors may be important in attracting women to specialize in PH, although this did not emerge strongly in this study. The scarcity of part-time positions was a concern of some women respondents with young children. Women's preferences for part-time work must be considered in workforce planning for PH and other medical specialties, as this affects training numbers and the availability of part-time posts.

### Motivations

In SA, PHM is a specialty that is largely chosen by physicians who reflect on their clinical careers and experience and then decide to work in settings focused on improving the health status of populations. Most specialized to contribute to social change and to impact on health services, thus maximizing population level health outcomes and service quality. This motivation is consistent with findings from a SA PHM registrar study (10) and a New Zealand (NZ) study of PHM specialists (30). PH approaches are underpinned by values and concerns for social justice (31–34). The desire to impact on the health status of populations through advocacy, social interventions—such as the reduction of poverty, found elsewhere (35)—and working to improve health systems was a consistent feature of respondent's motivations.

Formative influences on career choices included specialist training experiences, mentors, personal aptitudes, and careers that suited domestic circumstance, and this was also found the UK where, like SA, clinical work is not part of the scope of practice (24). In countries where PH roles are combined with clinical work, physicians were motivated to study PH to obtain a more holistic understanding of health systems, and skills to integrate PH in clinical roles (36, 37).

A lengthy service experience and accompanying maturity, positions trainees to gain maximally from training programs, and prepares them to work in complex systems. Similar to an earlier study of physicians with MPHs (26), respondents in this study embarked on PHM training after substantial work experience (median 7 years vs. 8 for MPH physicians), and many already had PH or other graduate training prior to specialization. This accords with reports from NZ and the UK: in the UK, physicians began PH specialist training after an average 5 years of practice (24); and substantial practice experience is implied in a NZ study (30). Although in the past, Canadian entrants to PH programs had years of clinical experience, and may in Australia still do, currently, in Canada (38) and Australia (39), many embark on training early in their careers.

In our study, many went on to further studies such as PhDs and this is also found elsewhere for example, in Italy (28). Although the need for continuing PH education for PH specialists is advocated for the PH workforce globally (40), this was not raised by respondents in our study. It is, however, highlighted in current Australian literature (39), and is required in the USA and Canada (41).

### Training Programs

Respondents valued the combination of experiential and formal training, which developed their skills in management, policy making, leadership, and research, competencies core to PH professionals working at senior levels. Nevertheless, the content and depth of training programs depended on individual universities and were uneven, as was found in French training programs (17).

In this study, specialists studied as trainees (registrars) alongside graduates from a range of disciplines enrolled in MPH programs, management, and occupational health diplomas. This exposure may have sensitized them to the importance of other PH professionals and could underlie their responses regarding the roles of other PH professionals, who were seen to contribute to the full range of PH roles. This highlights the recurring global and historical theme of the multi-disciplinary nature of PH (21, 42) and its interdisciplinary workforce (43).

A 4-year period of service placements, a key difference between registrar and MPH training, enabled practice learning and the development of competence in core areas. Competence in postgraduate medical education requires that students demonstrate ability and skill, which demands ongoing work-based assessment, formative feedback, and learner self-directed assessment (44). Competency-based PH curricula, aligning education, and work, are being promoted globally (45–47). Training must be focused, with close supervision, and tailored to registrars' interests in a wide range of settings.

Consistent with a number of other studies, including a study of South African PHM registrars (10), PH practice in the UK (48), the USA (49), and Switzerland (50), as well as field epidemiology masters' programs in LMICs (51, 52), specialists in this study valued competencies in epidemiology, surveillance, and communicable disease control. They highlighted placements in hospital management, health informatics, and disease programs, which developed their skills in project management, policy analysis, and planning as core PH competencies. This focus is also found globally, for example, in the USA (53), Brazil (54), and LMICs (55). Management skills, a scarce skill in SA (6, 56). are critical to health systems improvement, which many PHM registrars desired (10). In SA, PHM educators need to engage with initiatives (57) to develop much needed management competence, and PHM training programs need to prioritize these skills.

Most respondents conducted service-related research projects during their training. Designing and conducting research, followed by report-writing and oral presentations, all hone professional autonomy, critical and synthetic thinking as well as writing skills—core attributes contributing to a versatile PHM specialist. Health systems research, core to PH globally (58), for health system reform (59), underpins systems strengthening (49). While the health service research projects of PHM specialists in training, focusing on the relationships between service delivery and the health needs of populations (60). may be narrower than health systems research which addresses the building blocks of a health system (61), they can provide information for local decision-making and demonstrate the value-add of PHM specialists. Thus, well-trained PHM specialists are prepared for, can participate in and lead health services and systems research at all levels of the services.

### Public Health Practice

The sizable proportion (20%) who had not registered as specialists suggests that specialist registration was not unanimously viewed as advantageous. The career advantage of specialist training was raised by SA PHM registrars (10) and was debated in Canada in 2013 where specialty certification is no longer required for MOH positions (13). Board certification is reason for training in many countries including the USA (53), and many respondents valued being a registered specialist. Yet the career and job advantage of the specialty is ill-defined and needs clarity in SA.

For most (95%), PHM training influenced career choices, which was also found in reviews of postgraduate programs in the UK (24). Training gave direction to careers, and opened up work opportunities. Despite their long careers (median = 15.5 years), surprisingly few had multiple job changes (median of 3 jobs; 21% had only one job). Consistent with Canada (13), Italy (28), and NZ (30), their practice has largely been in the state health sector and academic environments. In 2010, most (70%) worked either in the state health sector or at universities, as managers, academics, or researchers. Health services careers were also found to be the work of PH physicians in Canada, France, Japan and the USA (17), and in Italy, one third of health managers are PH trained physicians (28).

A high proportion of PHM specialists (38.8%) also worked as researchers or in universities. Although research and academic work is a noted career destination for PHM specialists in Canada (13), France (17), NZ (30), Italy (28), and the USA (17) this may point to a respondent bias or to SA contextual factors, such as training programs that orientate graduates to research and academic environments, as well as the non-availability or unattractiveness of positions in the health services.

### Retention in the Public Sector

Many (72%) left the public sector at some point due to frustration with this environment, looking for new challenges, and some moved to academic work. Only half who had ever worked in the public sector had stayed. While some movement between employers is normal and enables people to find work niches, the large proportion who left the public sector, citing job dissatisfaction, and the unidirectional trend in movement out of the public sector, is concerning. These concerns are reflected in a Japanese study, where the retention of younger PH physicians in the public sector dropped from 73% between 1994 and 96 to 50% between 2004 and 6 (62). In the USA, PH professionals who were not satisfied with their work were 3.5 times more likely to consider leaving (22).

Retention of skilled staff is key to a stable, productive workforce, and reasons underlying high staff turnover needs to be addressed. USA research shows that changes in organizational and supervision arrangements facilitated PH workforce recruitment (22). Retention of scarce doctor skills in rural Australia hinged on remuneration, workplace organization, personal recognition, and social support (63), and these, together with study opportunities and mentorship strategies, were ways to retain physicians in rural Ghana (64).

Although a large proportion (73%) would consider a move to the state sector, remuneration alone would not attract specialists and employers would need to value innovation and reform to attract and retain them. In the USA, a study on PH workforce retention found that pay, whilst being important for retention, was less important than job satisfaction (22).

### Job Satisfaction

The global measures used to elicit respondents' job satisfaction encompassed overall attitudes toward work, but did not measure intrinsic, extrinsic, and demographic factors which are sometimes included in many job satisfaction studies (65). Intrinsic factors such as autonomy (66) and work content (67), and extrinsic factors such as professional development, and recognition (68), are predictors of work satisfaction in studies on nurses. A limitation of this study was being unable to drill down to these components. Identifying factors that improve job satisfaction for this cadre of skilled professionals may point to factors important for retention.

Nonetheless, PHM specialists, both men and women, reported they were largely fulfilled in their work and 41% had “found their niche.” This is expected as they occupied managerial, academic, and technical positions which are likely to be characterized by high levels of work autonomy and a sense of achievement, key factors for work satisfaction. Older respondents were 2.8 times more likely to be highly satisfied than younger ones, which is consistent with higher job satisfaction rates found amongst older health professionals reported in other studies (69, 70). This may be due to their job seniority, responsibility, and autonomy, that usually increase with age and are associated with higher job satisfaction (71). Many reported further education made possible by their jobs, and education options with promotion has predicted job satisfaction in studies among academics (65) and nurses (64).

### The Public Health Medicine Specialty

PHM's extensive apprentice type service learning coupled with theoretical training enables the fast-tracking of competent professionals ready to take on leadership and management responsibilities at senior levels in the health sector. This accords with senior leadership roles in studies of PHM specialists' roles in the UK (72), Canada (13), and NZ (30). Leadership is a much needed PH competency globally (49)—in high- (72–74), middle- and low-income (75, 76) countries.

Surprisingly, in view of the few positions identified for PHM specialists, specialists did not view other PH professionals as competing with them for employment, probably as respondents were professionally well-established. Indeed, many helped set up MPH programs nationally. However, they believed that specialists' current roles and careers were uncertain, and that the specialty was unappreciated and overlooked. Concerns about recognition, also found among registrars in South Africa (10), may be due to SA's contextual factors. These include the small size of the PHM pool, and that many work in non-specialist management posts, both of which contribute to the invisibility of the specialty. Concerns about recognition and roles were highlighted in a recent six country study of PH physicians (17). Underlying these concerns are questions about PH physician specialist work/professional boundaries compared to other PH professionals (33, 46, 77) and their professional identity (30), which were also articulated by some respondents.

### Public Health Medicine's Future

As a resource for much needed management, technical, and research skills as well as training capacity, the specialty had much to offer SA's reforming health sector. In view of the policy moment for health reform in SA, honing competencies to match the policy need is important for the sustainability of the specialty. Despite advocating for the inclusion of PHM specialists in provincial and district health establishments, outside of two provinces reported, no further positions have been created. Consequently, consistent with respondents' recommendations, in 2018, the CPHM held regional discussions about specialists' roles and competencies with stakeholders such as national and provincial health departments, academic trainers, and registrars and are finalizing these. To contribute to health systems strengthening and ensure its sustainability, CPHM needs to advocate for the profession and identify possible PH career pathways (39), which could result in the creation of jobs which, in turn, has implications for training programs. Accordingly, in August 2019, the CPHM submitted a motivation for the inclusion of PHM specialists and PH units at provincial and district levels to national policy-makers tasked with devising the next 10-year HRH plan.

### Limitations of the Study

We utilized three databases for our study—the HPCSA register of specialists, the CPHM database of successful examination candidates and the various universities training departments' databases. We may have omitted specialists not on these databases, but this is likely to be a handful. The research was conducted in 2012 and could not include more recent graduates' experiences, a limitation of the study. However, another study, conducted at the same time, of specialists-in-training—registrars—who went to qualify after 2012 (10) found that their perceptions about the specialty accorded closely with the specialists in this study. Moreover, the employment milieu for PHM specialists in SA has not shifted since 2012, and there are still few PHM specialist posts in the health services. The proportions of specialists registering with the HPCSA is likely to be significantly higher than the 42% found in this study as the OSD incentivizes early specialist registration. Salaries are largely dependent on the years working as specialists post HPCSA registration.

Although the response rate was 67% with a survey completion rate of 56%—a percentage close to the 60% acceptable threshold for online surveys (78)—selection biases, in different directions, may be present. Those who elected to participate may feel more positively about the profession and its potential to affect the services in an era of health services reform than non-responders; alternatively, they may have been the dissatisfied who welcomed the opportunity to vent. The large proportion of university-based respondents may over-represent this career option. These specialists were easier to contact, may be more likely to respond to research requests given this is core to their work, and the lead researcher on the project may have been known to them. To check for a selection bias, comparisons between those who responded and the overall database found similar proportions for sex, undergraduate and specialist training institutions; the median year of specialization, but respondents exited medical training on average three later than the year of exit for the whole database, meaning they were more recent graduates. This may imply that the experiences, career paths and job satisfaction of earlier specialists are incompletely represented.

The restricted options on the quantitative instrument may have limited responses. For example, many remarked in open-ended questions that PHM's unique contribution was its breadth of competencies, but this was not asked directly. Similarly, we did not ask about the factors that affect their job satisfaction. The use of questionnaires to probe perceptions or opinions can result in shallow data and qualitative methods such as interviews may have been better suited.

## Conclusions

This study is the first known to explore the motivations and careers of Public Health Medicine specialists in a LMIC such as SA. Despite PHM in SA being a small medical specialty, specialists are widely distributed in different settings. As is found elsewhere, they work in health service and university settings as managers, academics, and researchers. Their work is focused on population health, largely motivated by a commitment to social justice as well as their interest in specific content areas, and they are largely satisfied in their work. However, as elsewhere (17), PHM is challenged by uncertainty about its scope, uniqueness, recognition, and career paths within SA's health system.

A range of PH professionals are needed in SA, many of whom are better suited to perform critical technical functions than medical specialists. As was argued by the Welsh Faculty of PH (77), not all specialists could be expected to have the same depth of skills. PHM specialists, however, offer a unique combination of skills preparing them for a range of positions including senior health leadership positions. PHM competencies include generic skills such as communication (79), leadership, management, critical appraisal, and synthesis skills (39) which together with specialized skills in epidemiology are required to transform the health services and address priority health needs. However, unless work environments encourage efficiency, autonomy, professional development, and leadership, improved remuneration alone will not attract physicians to specialize and work in the state health sector.

In SA, the specialty is undervalued and not sufficiently inserted into the design of health sector structures—both service delivery and health agencies. It is much more strongly located in academic departments in universities.

SA's health sector is highly resource constrained and dominated by curative imperatives. PHM is therefore challenged to demonstrate its value through (1) the work of its trainees who are present in many provincial services, (2) specialists who work in managerial and academic positions and in (3) training and research. The proactive approach the CPHM has embarked on, engaging a range of policy making stakeholders, to refine core competencies, appropriate training and work niches, should contribute to advocacy for a revitalized specialty that will contribute to current health reform in SA. Our SA experience can contribute to debates internationally and on the African continent (80) about appropriate curricula and career paths for specialists whilst at the same time acknowledging the multidisciplinary nature of PH practice and the broad church of her professionals.

## Data Availability

The datasets generated for this study will not be made publicly available as consent taken for participation assured informants anonymity and confidentiality.

## Ethics Statement

The studies involving human participants were reviewed and approved by University of Cape Town's (UCT) Faculty of Health Sciences Human Research Ethics Committee. The patients/participants provided their written informed consent to participate in this study.

## Author Contributions

VZ conceptualized the research, conducted the fieldwork, analyzed the data, and drafted the article. Together with other studies, this research formed part of her doctoral studies. LL supervised the research, data analysis, and contributed to the final article. WP supervised the research and contributed to the final article.

### Conflict of Interest Statement

The authors declare that the research was conducted in the absence of any commercial or financial relationships that could be construed as a potential conflict of interest.
