# Supplementary material for: Career Paths of Public Health Medicine Specialists in South Africa
Source: Front Public Health. 2019 Sep 12;7:261. doi: 10.3389/fpubh.2019.00261 (PMC6751254; doi:10.3389/fpubh.2019.00261)
Supplement: Supplementary file 1 [file Data_Sheet_1.docx]

**QUESTIONNAIRE**

***Please complete as fully as you can.***

1 Age: ....................

2 Gender: ...............

**UNDERGRADUATE training**:

3 Undergraduate degree: ...................

4 Institution obtained: ...........................................................

5 Year undergraduate degree obtained: ......................

**POSTGRADUATE Public Health or Community Health training**:

6 Post graduate public health training institution: ................................................

7 Years when trained: ........................

8 Rotations:

|  | **rotation 1** | | **rotation 2** | **rotation 3** | **rotation 4** | **rotation 5** | **rotation 6** |
| --- | --- | --- | --- | --- | --- | --- | --- |
| **Place** |  | |  |  |  |  |  |
| **Time (e.g. 1 year)** |  | |  |  |  |  |  |
| **Role** |  | |  |  |  |  |  |
| How would you rate the rotation?  1=poor (frustrating),  2= OK experience, but no real added value  3= useful (gave useful insight);  4=good learning experience (use skills developed on a daily basis);  5=invaluable and gave direction to career choice | | | | | | | |
| **Rating** | |  |  |  |  |  |  |

9 Other comments on usefulness of rotations: ,,,,,,,,,,,,,,,,,,,,,,,,,,,,,,,,,,,,,,,,,,,,,,,,,,,,,,,,,,,,

....................................................................................................................................

....................................................................................................................................

....................................................................................................................................

10 Additional information about training (e.g. supranumerary status, switching training institution) that you would like to make. .................................................................

....................................................................................................................................

....................................................................................................................................

....................................................................................................................................

11 Formal courses completed prior to entry on registrar programme:

a) ...........................

b) ...........................

c) ...........................

12 Formal courses completed during registrar training (e.g. MPH (no of modules); DOH):
a) ...........................

b) ...........................

c) ...........................

d) ...........................

13 Qualification obtained (circle all applicable): MMed / Fellowship /Other
 Give detail: .........................................

14 Did you register your qualification with the HPCSA? Yes / No

Outline reason for doing this: ...................................................................................

....................................................................................................................................

15 Did you register as a Public Health Medicine / Community Health specialist with the HPCSA? Yes / No

Outline reason for doing this: ...................................................................................

....................................................................................................................................

16 Have you undertaken additional formal studies after qualifying as a specialist: Yes / No
 Give detail (e.g. PhD, Masters, MBA) .....................................................................

....................................................................................................................................

**WORKING LIFE**

17 Work post qualification (please complete table and use additional space if necessary)

| **institution** | **position** | **dates** | **reasons for staying** | **reasons for leaving** |
| --- | --- | --- | --- | --- |
|  |  |  |  |  |
|  |  |  |  |  |
|  |  |  |  |  |
|  |  |  |  |  |
|  |  |  |  |  |

18 What would you say were the key **drivers** in determining your career trajectory so far?: ....................................................................................................................................

....................................................................................................................................

....................................................................................................................................

19 In what ways would you say that your public health medicine **training** contributed to your specific career direction within Public Health?

....................................................................................................................................

....................................................................................................................................

....................................................................................................................................

20 Are there aspects of your training that you would have liked to use but were unable to? Give detail.

....................................................................................................................................

....................................................................................................................................

21 Are there areas in your work that you think your Public Health / Community Health training could have equipped you better to deal with?:

...................................................................................................................................

....................................................................................................................................

....................................................................................................................................

22 Would you say that you are happy with your career trajectory thus far?

| 1=unhappy | 2= OK | 3 = satisfied | 4 = pleased | 5= fulfilled |
| --- | --- | --- | --- | --- |

Please explain: ...........................................................................................................

....................................................................................................................................

23 If you worked in the Public Sector after specialisation how would you rate your experience?

| 1=frustrating/deskilling | 2= OK | 3 = mixed experience | 4 = good use of skills | 5= good use of skills + impact |
| --- | --- | --- | --- | --- |

Please explain: ..........................................................................................................

....................................................................................................................................

24 If there were advertised Public Health Medicine (PHM) positions within the Public Sector health services, would have you considered this as a longer-term career option?

| 1=never | 2= maybe | 3 = definitely |
| --- | --- | --- |

Give detail.

....................................................................................................................................

....................................................................................................................................

....................................................................................................................................

25 Do you think that the recently implemented Occupational Specific Dispensation (OSD) for doctors would attract PHM specialists into the Public Service? Yes / No / Don’t know

Please explain ..........................................................................................................

....................................................................................................................................

....................................................................................................................................

26 In the following areas, what do you see the potential role for Public Health Medicine / Community Health specialists in the Public service as being?

1. Strategic planning:

| 1 = wasted resource | 2 = not needed | 3 = useful | 4 = added value | 5 = critical |
| --- | --- | --- | --- | --- |

Explain.........................................................................................................................

....................................................................................................................................

1. Programme design:

| 1 = wasted resource | 2 = not needed | 3 = useful | 4 = added value | 5 = critical |
| --- | --- | --- | --- | --- |

Explain.........................................................................................................................

....................................................................................................................................

1. Epidemiology:

| 1 = wasted resource | 2 = not needed | 3 = useful | 4 = added value | 5 = critical |
| --- | --- | --- | --- | --- |

Explain.........................................................................................................................

....................................................................................................................................

1. Surveillance:

| 1 = wasted resource | 2 = not needed | 3 = useful | 4 = added value | 5 = critical |
| --- | --- | --- | --- | --- |

Explain.........................................................................................................................

....................................................................................................................................

1. Health impact assessment:

| 1 = wasted resource | 2 = not needed | 3 = useful | 4 = added value | 5 = critical |
| --- | --- | --- | --- | --- |

Explain.........................................................................................................................

....................................................................................................................................

1. Management (hospital, other line, programme):

| 1 = wasted resource | 2 = not needed | 3 = useful | 4 = added value | 5 = critical |
| --- | --- | --- | --- | --- |

Explain.........................................................................................................................

....................................................................................................................................

1. Other: .........................................................................................................................

....................................................................................................................................

27 What suggestions do you have for improving the career paths of PHM specialists in South Africa?

....................................................................................................................................

....................................................................................................................................

....................................................................................................................................
